# *Salvinia*-like slippery surface with stable and mobile water/air contact line

**DOI:** 10.1093/nsr/nwaa153

**Published:** 2020-07-02

**Authors:** Xiaomei Li, Jinlong Yang, Kaixuan Lv, Periklis Papadopoulos, Jing Sun, Dehui Wang, Yanhua Zhao, Longquan Chen, Dapeng Wang, Zuankai Wang, Xu Deng

**Affiliations:** Institute of Fundamental and Frontier Sciences, University of Electronic Science and Technology of China, Chengdu 610054, China; Institute of Fundamental and Frontier Sciences, University of Electronic Science and Technology of China, Chengdu 610054, China; State Key Laboratory of Polymer Physics and Chemistry, Institute of Applied Chemistry, Chinese Academy of Sciences, Changchun 130022, China; Physics Department, University of Ioannina, Ioannina 45110, Greece; University Research Center of Ioannina (URCI), Institute of Materials Science and Computing, Ioannina 45110, Greece; Department of Mechanical Engineering, City University of Hong Kong, Hong Kong, China; Institute of Fundamental and Frontier Sciences, University of Electronic Science and Technology of China, Chengdu 610054, China; Department of Mechanical Engineering, City University of Hong Kong, Hong Kong, China; School of Physics, University of Electronic Science and Technology of China, Chengdu 610054, China; State Key Laboratory of Polymer Physics and Chemistry, Institute of Applied Chemistry, Chinese Academy of Sciences, Changchun 130022, China; Department of Mechanical Engineering, City University of Hong Kong, Hong Kong, China; Institute of Fundamental and Frontier Sciences, University of Electronic Science and Technology of China, Chengdu 610054, China

**Keywords:** slippery Cassie state, *Salvinia molesta*, stability, low adhesion, drag reduction

## Abstract

Superhydrophobic surfaces are widely used in many industrial settings, and mainly consist of rough solid protrusions that entrap air to minimize the liquid/solid area. The stability of the superhydrophobic state favors relatively small spacing between protrusions. However, this in turn increases the lateral adhesion force that retards the mobility of drops. Here we propose a novel approach that optimizes both properties simultaneously. Inspired by the hydrophobic leaves of *Salvinia molesta* and the slippery *Nepenthes* pitcher plants, we designed a *Salvinia*-like slippery surface (SSS) consisting of protrusions with slippery heads. We demonstrate that compared to a control surface, the SSS exhibits increased stability against pressure and impact, and enhanced lateral mobility of water drops as well as reduced hydrodynamic drag. We also systematically investigate the wetting dynamics on the SSS. With its easy fabrication and enhanced performance, we envision that SSS will be useful in a variety of fields in industry.

## Teaser

Inspired by the hydrophobic leaves of *Salvinia molesta* and the slippery *Nepenthes* pitcher plants, we designed a *Salvinia*-like slippery surface consisting of protrusions with slippery heads.

## INTRODUCTION

Superhydrophobic surfaces (SHPOS), mainly inspired by natural surfaces, have captured great interest in both fundamental and applied research and industry. Possible applications include self-cleaning, antifouling, anti-icing and drag reduction applications [1–7]. SHPOS consist of a solid porous framework filled with an air cushion, which is crucial to maintain the Cassie wetting state and liquid repellency. The air cushion minimizes the liquid/solid contact area, thus a water drop on SHPOS shows high contact angle and small roll-off angle. However, the completely wetted Wenzel state, without air cushion, is usually thermodynamically more stable. Extensive efforts have been made to stabilize the air cushion inside the surface, mainly focusing on optimization of the morphology of the structure [8–11]. However, optimization of the stability of the Cassie state (stable microscopic air/water contact line in the vertical direction) requires large protrusions with small spacing, in contrast to the minimization of the lateral adhesion force (laterally mobile air/water contact line) which requires the opposite [12]. Thus, the simultaneous optimization of both remains a great challenge for SHPOS with high performance.

**Figure 1.**
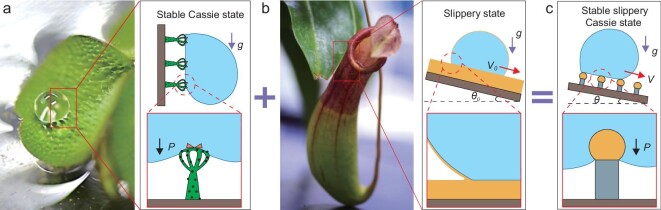
Design of the SSS. (a) *Salvinia molesta* floating leaf on which water drop displays stable Cassie state. Although the rational design of elastic eggbeater-shaped microstructure with surface energy gradient in vertical direction could stabilize the contact line to prevent impalement, such structure has strong adhesion because of the hydrophilic patches. (b) A pitcher plant-inspired slippery surface with molecularly smooth lubricant fixed on the top of the microstructure, which enables fast drop or liquid transportation. (c) The combination constitutes the SSS on which water drop shows slippery stable Cassie state. The black, purple and red solid arrows represent the directions of pressure (*P*), gravity (*g*) and the velocity of drop transport (*V*), respectively.

On another research line, slippery liquid-infused porous surfaces (SLIPS) have been demonstrated as promising substrates where low lateral adhesion force for drops of any liquid is required [13]. These were inspired by the *Nepenthes* pitcher plants that are covered with a water-based lubricant and allow drops to slide down easily with low contact angle hysteresis (Fig. 1b). Artificial surfaces usually employ fluorinated or other immiscible lubricants. A drop on a liquid-infused slippery surface, however, shows both smaller contact angle and shedding velocity compared to the SHPOS. The introduction of a stable air cushion between protrusions with slippery surface is challenging because of the low surface tension of the lubricant [14]. Recently, attempts to combine SHPOS and SLIPS using doubly re-entrant microstructures with a slippery lubricant-infused layer on top, were employed to prevent drop impalement inside the air cushion [15].

*Salvinia* leaf has attracted the attention of researchers because of its long-lasting Cassie state under water [16,17]. Such a stable Cassie state was attributed to the hydrophobic eggbeater-like trichomes with hydrophilic pins on top. The hydrophilic-to-hydrophobic boundary pins the water/air contact line in the vertical direction and supports a stable Cassie state for the drop [16] (Fig. 1a). However, the pinning effect also increases lateral adhesion forces and diminishes the mobility of the contact line at the horizontal direction.

Herein, inspired by the *Salvinia* leaf with stable water/air contact line and *Nepenthes* pitcher plants with mobile water/air contact line, we constructed a *Salvinia*-like slippery surface (SSS) that synergistically combines both preferential properties. Using a lubricant-infused cross-linked polydimethyl siloxane (PDMS) layer on the top of pillars with hydrophobic side walls, drops on the SSS show stable slippery Cassie state, avoiding the strong pinning effect on the hydrophilic pitches of the *Salvinia* plant (Fig. 1c). We show that the Cassie state on SSS is more stable against drop evaporation and impact with respect to a control surface with the same structure without lubricant. The low liquid/solid adhesion is also proven by measurements of lateral forces during horizontal drop motion. We envision that our design will be useful in a variety of applications requiring drag reduction of fluids.

## RESULTS AND DISCUSSION

### Design of the SSS

**Figure 2.**
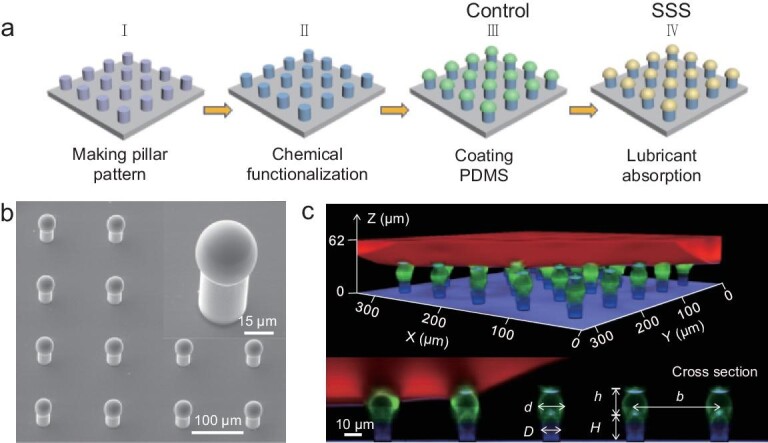
Fabrication process and topological structure of the SSS. (a) Combination of hard and soft lithography. Micropillars are first made of the SU-8 photoresist and then coated with PDMS hemispheres. (b) ESEM images taken at an angle of 45° showing high regularity and structural details of the SSS (*d* = 36 ${\rm{\mu m}}$, *D* = 25 μm, *b* = 120 μm, *h* = 33 μm, *H* = 45 μm). (c) Three-dimensional confocal microscopy image of a drop on the SSS and its cross-section (*d* = 15 ${\rm{\mu m}}$, *D* = 10 ${\rm{\mu m}}$, *b* = 60 μm, *h* = 16 μm, *H* = 20 μm). Fluorescent emissions from water, oil-infused PDMS and SU-8 pillars are shown in red, green and blue, respectively. Reflection is shown in purple.

Several strategies have been developed for engineering oil-infused slippery surfaces [13,18,19]. Here, we used silicone oil-infused crosslinked PDMS film, reported to be a slippery, defect-free and long-lasting lubricated layer [20]. The SSS was composed of an array of hydrophobic micropillars, the top of which was decorated with an oil-infused PDMS sphere (Fig. 2a) that serves as the slippery surface. Micropillar arrays were first fabricated by photolithography, followed by fluorination to reduce the surface free energy. To generate the spherical PDMS top on the micropillars, a thin PDMS film was prepared on a cover glass by spin coating and then transferred onto the top of the hydrophobic micropillars by a dip-coating technique (see Methods) [21,22]. PDMS formed spheres as a result of surface tension. After fully curing, a mushroom-like SHPOS was formed (control surface). Finally, the control surface was immersed into a silicone oil bath for 48 hours to absorb silicone oil. After taking out the surface from the oil bath and extracting excess silicone oil from the inter-micropillar gap using toluene, we got the SSS. The high uniformity and regularity of the SSS and the control surface are shown in environment scanning electron microscopy (ESEM) and scanning electron microscopy (SEM) images (Figs 2b and S1). Confocal imaging showed that water drops sitting on the surface form a stable Cassie state (Fig. 2c). It should be noted that the PDMS sphere on top of the micropillars also serves as a re-entrant structure, which could better stabilize the Cassie state for both the control surface and the SSS, compared to a simple array of micropillars with vertical walls [10].

To compare the wetting properties of the control surface and the SSS, we measured the apparent contact angles and roll-off angles of water drops on both surfaces with different solid fractions (Fig. S2), respectively. Drops on the SSS had a smaller roll-off angle than that on the control surface at the whole range of solid fractions (Fig. S2c), demonstrating that the silicone oil on the PDMS acted as a lubricant and made the SSS slippery.

### Lateral mobility of the microscopic water/air contact line on the SSS

**Figure 3.**
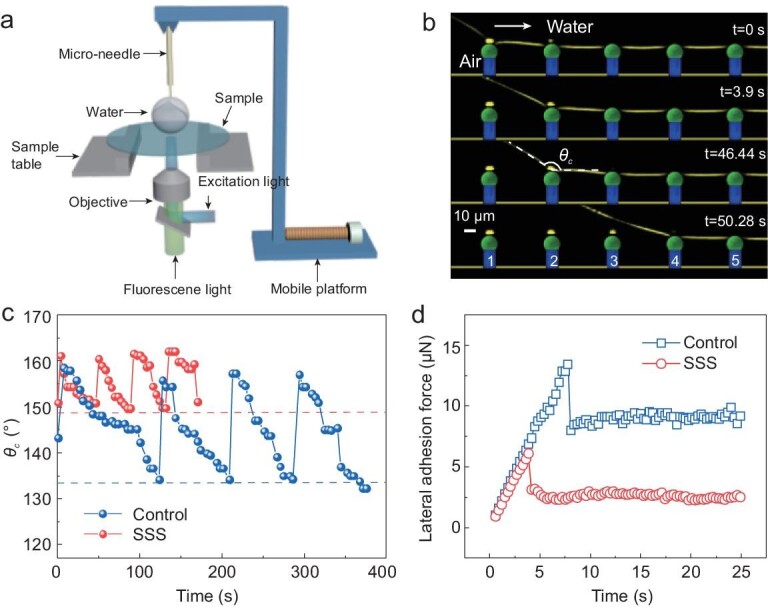
Lateral pinning and adhesion forces on the SSS. (a) Schematic of the experimental setup. The micro-needle moved at 0.01 mm/s and its deflection was used to measure force. The confocal microscope was used for imaging. (b) Confocal vertical cross-section of the receding side of a water drop moving to the right on the SSS. Colors as in Fig. 2c. (c) Apparent ${\theta _c}$ during needle movement. When the receding angle value was reached (dashed line), the contact line jumped. (d) Measured lateral adhesion force of water drop on the control surface and the SSS with time. The dimension is: *d* = 15 μm, *D* = 10 μm, *b* = 60 μm, *h* = 16 μm, *H* = 20 μm.

The manifestation of smaller roll-off angles on the SSS relative to control surfaces proves that the utility of slippery PDMS spheres serves to improve drop mobility. We further quantified the mobility by manipulating drops on both types of surface and measuring the shape of the drop with confocal microscopy, or lateral adhesion forces with a cantilever. In both cases a microneedle controlled by a motorized stage was used to move the drop at constant velocity (Fig. 3a, Video S1). We measured vertical slices through the left side of the drop with the confocal microscope in simultaneous reflection and fluorescence mode (Fig. S3a). The images show the micropillars and the water/air interface (Fig. 3b). By moving the drop to the right, the macroscopic contact line, i.e. in length scale larger than the micropillars, started to recede. Confocal microscopy reveals the details of this motion with resolution much higher than the size of micropillars. The actual motion of the microscopic contact line is discontinuous (Figs 3b and S3b). The contact line was pinned until the critical receding macroscopic contact angle ${\theta _c}$ was reached and then jumped to the next pillar. To quantitatively compare the contact line receding procedure between the SSS and the control surface, we calculated ${\theta _c}$ just before each jump. The value of ${\theta _c}$ on the SSS was always larger than that of the control surface (Fig. 3c). It is known that the macroscopic receding contact angle is related to adhesion forces and the roll-off angles. Conforming to the continuum mechanics models [23], for a drop moving quasi-statically on a solid surface, the lateral adhesion force ${F_r}\ $can be predicted as

(1) \begin{equation*} {F_r} = \ kR{\gamma _{LV}}\!\!\left( {\rm {\cos}{\theta _R} - \rm {\cos}{\theta _A}} \right), \end{equation*}

where ${\gamma _{LV}}$ is the surface tension of water, *k* is a factor in the order of 1 that depends on the shape of the drop, *R* is the macroscopic contact radius of the drop, and *θ_A_* and *θ_R_* denote the macroscopic advancing and receding contact angles, respectively. As the advancing angles are almost equal to 180° on surfaces composed of micropillars [24], the different receding contact angle values on the SSS (150°) and control surface (134°) suggest that a water drop on the control surface needs to overcome about twice the force on the SSS. Therefore, drops on the SSS have a higher mobile contact line.

To directly measure the lateral adhesion forces under static and dynamic conditions, we used the microneedle as a cantilever-based force sensor [25] (Fig. S4a, Video S2, Supplementary methods). The results (Fig. 3d) show that both the static force and the kinetic force of the SSS (6 μN and 3 μN) were smaller than those of the control surface (14 μN and 9 μN), by about two and three times, respectively. Adhesion forces depend on the area fraction of micropillars, the drop size and drop moving velocity, but were always lower on SSS compared to the respective control surface, showing that the effect is universal (Fig. S4b–d). This is expected, as roll-off and adhesion forces are both related to the force exerted by each pillar to the drop. The reduction of adhesion forces is important for applications, as it means lower energy consumption for moving drops.

### Stability of the Cassie-Baxter wetting state on the SSS

**Figure 4.**
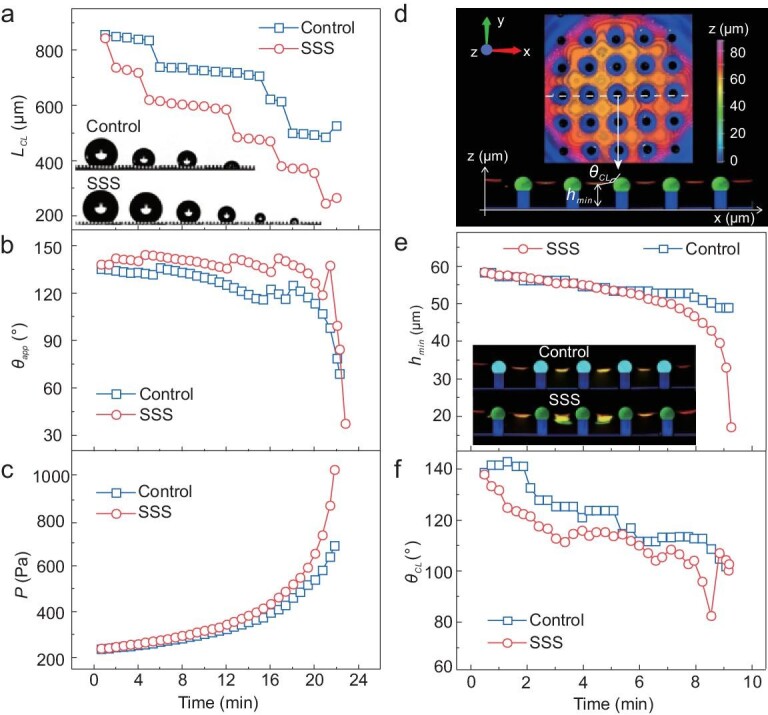
Stability of the Cassie state on the SSS during evaporation. (a) Change of the contact base diameter of water drop *L_CL_* with time on the control surface and the SSS (the insets are the selected snapshots, obtained with a high-speed camera, of water drops evaporating on the control surface and the SSS). (b) Apparent contact angle ${\theta _{app}}\ $and (c) Laplace pressure of a water drop on the control surface and the SSS. (d) Confocal image of the reflection from the water/air cushion interface of a drop deposited on the surface and its X-Z cross-section along the white dotted line. We extract the microscopic contact angle *θ_CL_* and the minimum height of the air cushion ${h_{min}}$. Micropillars have been reconstructed with dimensions known from SEM measurements. (e) Thickness of the water/air cushion ${h_{min}}$ on the control surface and the SSS with time (the inset shows profiles of the air cushion through the center of the water drop, taken every 6 s). (f) Microscopic contact angle *θ_CL_* on the control surface and the SSS with time (the intrinsic angles of the surface showed in Table S2). The dimension is: *d* = 36 μm, *D* = 25 μm, *b* = 120 μm, *h* = 33 μm, *H* = 45 μm.

Figure 2c shows that drops deposited on the SSS are in the Cassie-Baxter wetting state, characterized by the trapping of an air cushion within rough surfaces. It is known, however, that a transition to the completely wetted Wenzel state can be triggered by an increase in pressure [26,27]. To investigate whether SSS also stabilizes the Cassie state, we increased the Laplace pressure of drops by controlling the evaporation. To capture the macroscopic details of the transition, we used a high-speed camera to image the drop from the side. Two processes were observed in the course of evaporation on both SSS and the control surface: lateral stepwise depinning and, finally, impalement (Fig. 4a and b, Video S3). During the lateral depinning process, the macroscopic contact line depinned stepwise from the micropillars, similar to drops manipulated with the needle. As evaporation proceeded, the apparent contact angle$\ {\theta _{app}}$ (defined in Fig. S5) decreased until it reached the lowest possible value, the receding contact angle. Then the macroscopic contact line jumped to the next row of micropillars and the contact base diameter *L_CL_* decreased by the value of pillar center-to-center distance *b* = 200 μm (Fig. 4a). The apparent contact angle of the SSS was always larger than that of the control surface before complete impalement (Fig. 4b), in agreement with the measurements of the receding contact angle with moving drops (Fig. 3c). At some points, drops on both SSS and the control surface impaled the structure and transitioned to the Wenzel state, as shown by the absence of the air layer in the last frames of Fig. 4a. The transition is also reflected at the decrease of apparent contact angles to almost zero, as the receding contact angle in the Wenzel state is much smaller than that in the Cassie state. To quantify the stability of the Cassie state against impalement, we calculated the Laplace pressure of the drops from their shape as ($p\ = \frac{{2\gamma }}{r}$). Here, *r* is the radius of curvature at the top of the drop profile. The results showed that the transition occurred when pressure reached 940 Pa on the SSS and 690 Pa on the control surface. Thus, the SSS can stand about 35% more pressure than that on the control structure (Fig. 4c).

To explain this increased stability against pressure, we investigated the transition to the Wenzel state on the microscale, by applying confocal microscopy to image the water/air interface (Video S4). The interface is clearly visible in the reflection mode (Fig. 4d). By analyzing 3D stacks of confocal image, we extracted the coordinates of the interface and thus the local height of the air cushion. As a result of the Laplace pressure, the interface is curved. The parameters relevant to the transition are the minimum height *h_min_* (Fig. 4e) and the microscopic contact angle *θ_CL_* (Fig. 4f), i.e. on length scale smaller than the size of the pillars, but close to the resolution of the confocal microscope. Both values are extracted by suitable image analysis (Figs S6 and S7). In general, the transition to the Wenzel state can be triggered either by sagging, that is the decrease of *h_min_* to zero, or depinning, that is the increase of *θ_CL_* to the advancing contact angle of the material and the sliding down of the microscopic three-phase contact line. Here the transition proceeded through the second mechanism in both cases. The increase of pressure led to an increase of curvature that affected *θ_CL_*. There is, however, also an important difference between the SSS and the control surface. On the control surface the transition occurred abruptly when *θ_CL_* reached a critical value. Then *h_min_* decreased to zero within milliseconds, and the air cushion disappeared. This is similar to the process observed on stuctures with pillars with vertical walls [26]. On the SSS, the transition was much more gradual. Before the complete disapperance of the air cushion, intermediate wetting states were observed, where the microscopic contact line was on the side of the micropillars and not on the PDMS hemisphere. The sliding down of the contact line took about 1–2 min to complete. Thus the SSS not only increased the critical impalement pressure, but also slowed down the transition. These findings imply that the oil used to infuse the PDMS hemispheres changes the microscopic contact angles and induces dynamic effects.

### Explanation of the enhanced stable superhydrophobicity of the SSS

Experimental results showed that the SSS has several advantages over the control surface: lower contact angle hysteresis, lower roll-off angles, higher critical impalement pressure and slower Cassie-to-Wenzel transition. In this section we try to explain the observed effects, both experimentally and theoretically. The re-entrant structure enhances superhydrophobicity compared to vertical pillars, but is the same for both the SSS and the control surface. Thus, the differences must result from the presence of lubricant. Liquids that swell crosslinked polymers not only stay in the interior, but also form a thin layer on the exterior. Therefore, we investigated how the presence of a thin liquid layer on the PDMS hemispheres affects the microscopic contact angle.

**Figure 5.**
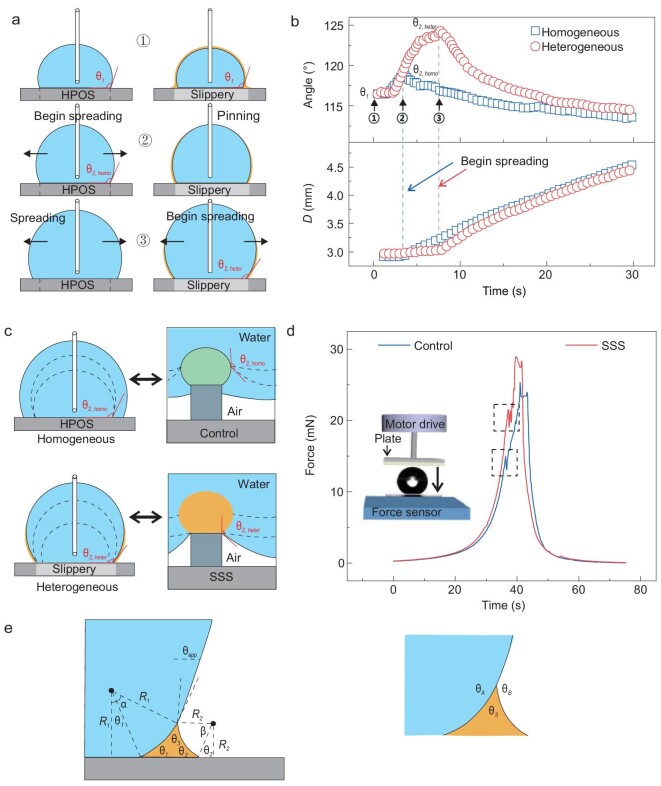
Contact line barrier at the contact line between a slippery surface and a hydrophobic surface (HPOS). (a) Schematic of a water drop spreading on a chemically homogeneous hydrophobic surface and a heterogeneous hydrophobic surface with a circular slippery surface at the center. A drop with a volume of 13 μL was deposited on the surface (circle 1) and expanded until the critical advancing contact angle was reached for homogeneous hydrophobic surface (circle 2). By further injecting water, the drop on homogeneous hydrophobic surface started to spread while the contact line of drop on the heterogeneous surface kept still until a higher critical advancing contact angle was reached (circle 3). (b) The change of apparent contact angle and the diameter of water drop as a function of time during spreading on the homogeneous surface and the heterogeneous surface. Numbers correspond to (a). (c) Contact line moving from a macro perspective to a micro perspective. (d) Critical force exerted on the water drop when impalement happened on the control surface and the SSS (dashed rectangles is the position when the Cassie-to-Wenzel transition happened). The dimension is: *d* = 15 μm, *D* = 10 μm, *b* = 30 μm, *h* = 16 μm, *H* = 20 μm. (e) Theoretical model of a drop (cyan) surrounded by an oil ridge (yellow) on a flat surface (grey). *θ*_1_ and *θ*_2_ are the contact angle values of the oil on the two sides of the ridge, *θ_A_*, *θ_B_* and *θ*_3_ are the angles of the water/oil/air Neumann triangle, and *R*_1_, *R*_2_ are the radii of curvature of the water/oil and oil/air interfaces. Angles *α*, *β* define the position of the water/oil/air contact line.

Note that the thin liquid layer is not clearly visible on confocal images, as it is probably too thin to be resolved. Therefore, we used flat model surfaces with similar chemical properties to measure contact angles. The schematic of the experiment is shown in Fig. 5a. First, we tested whether there is a contact angle barrier between the oil-infused crosslinked PDMS layer and the hydrophobic pillar. Two flat surfaces were used to measure advancing contact angles of water: (a) a chemically homogeneous hydrophobic surface, as control, and (b) a heterogeneous slippery-hydrophobic surface with a circular slippery surface at the center, which simulates the boundary between oil-infused PDMS hemispheres and the vertical micropillar walls. The latter consisted of a slippery circle with $3 \pm 0.5{\rm{\ mm\ }}$diameter in the center of a fluorinated hydrophobic surface (see Methods). Then, we deposited a drop on the homogeneous surface and increased its volume. At the beginning, the contact line was fixed and the contact angle increased (process ①) until it reached a maximum value of 118° (process ②). Then, the drop started to spread (process ③). On the heterogeneous surface, we started measurements with a drop that covered completely the 3 mm-diameter circle. By increasing the volume, we observed a similar trend, but the respective maximum contact angle at point ② was higher. This is because the macroscopic contact line was pinned at the slippery-hydrophobic boundary (Fig. 5a and b, process ① and ②). At the critical apparent contact angle ${\theta _{2,heter}}$, the water drop started to advance and the apparent contact angle decreased to around ${\theta _a}$ (Fig. 5a and b, process ③, Video S5). Compared to the chemically homogenous hydrophobic surface, a drop on the heterogenous slippery-hydrophobic surface started to advance at a higher critical contact angle (${\theta _{2,heter}} > {\theta _{2,homo}})$ (Fig. 5b). For the homogenous surface, the contact line started to advance at a higher apparent contact angle than the intrinsic advancing angle, which can be explained by the static-friction-like inertia principle [28]. For the heterogeneous slippery-hydrophobic surface, the formation of a viscous oil ridge (Fig. S8) can also induce dynamic effects, which could further increase the barrier for water drop to advance on the hydrophobic surface. For our slippery sphere structure on the micropillars, such an energy barrier also exists (Fig. 5c). Considering a drop deposited on the control surface or the SSS in the Cassie state, the water/air interface underneath the drop is governed by a force balance in the vertical direction as described in Ref. [29]:

(2) \begin{equation*} F\ = \ - \gamma \!\left( {\pi D} \right)\rm{\cos} \left( \theta \right), \end{equation*}

where *θ* is the contact angle of the water/air interface, *D* is the diameter of the micropillars, *γ* is the surface tension and *F* is the excess force per micropillar. This force is proportional to the applied pressure for a fixed pillar-to-pillar spacing. The critical advancing contact angles on the macro homogenous surface (${\theta _{2,homo}}$) are smaller than the one on the heterogeneous surface (${\theta _{2,heter}}$). According to equation (2), the Cassie state collapses on the SSS at higher pressure than that on the control surface. To verify this experimentally, we used a superhydrophobic plate to apply the vertical force on a suspended drop to trigger the Cassie to Wenzel wetting transition. The experiment is similar to the evaporation of drops, but here the water volume was fixed and forces were measured directly by a sensitive force sensor (Fig. 5d, Supplementary methods). We found that the SSS can withstand near 50% more excess force than the control surface (19.8 mN and 13.15 mN, respectively), which is consistent with theoretical calculations. Thus, we confirm that the contact angle barrier contributed to a more stable Cassie state for the drop on the SSS. We also measured effect of viscosity, the surface tension of the lubricant oil and the structural size on the results, by applying different oils and surfaces with different solid fractions (Figs S9 and 10, the wetting properties of different oils are listed in Table S1). The results were in line with the above conclusions.

To better understand the origin of the contact angle barrier, we used a simple model to predict the experimentally measured contact angle values (Fig. 5e) [30]. We consider a water drop surrounded by a ridge of PDMS lubricant, assuming that gravity is negligible and that the height of the ridge is small compared to the drop. Thus, the Laplace pressure of the drop and all curvatures in the horizontal plane are also negligible, so the vertical cross-sections of the PDMS/water and PDMS/air interfaces are circular. We calculated the apparent contact angle of the drop, at a height above the water/oil/air three-phase contact line, for different substrates, namely a homogeneous fluorinated glass that simulates the micropillar walls, PDMS for the hemispheres on the SSS and the boundary between these two. All microscopic contact angles (*θ*_1_, *θ*_2_, *θ*_3_, *θ_A_*, *θ_B_*, see Fig. 5e) are defined by the interfacial tensions and measured experimentally. For PDMS spreads on water, *θ*_3_ = 0, *θ_Α_* = *θ_Β_* = 180°. Also, *θ*_1_, *θ*_2_ are sensitive to the substrate. The oil ridge has two interfaces, water/oil and oil/air, with different radii of curvature *R*_1_, *R*_2_, respectively, that are related through $\frac{{{\gamma _1}}}{{{R_1}}} = \frac{{{\gamma _2}}}{{{R_2}}}$, where *γ*_1_ = 48 mN/m and *γ*_2_ = 19 mN/m are the respective interfacial tensions. We note that the oil ridge has a negative Laplace pressure. From the height of the water/PDMS/air contact line, we get ${R_1}\! ( {\rm{\cos}{\theta _1} - \rm{\cos}\alpha } ) = {R_2}\! ( {\rm {\cos}{\theta _2} - \rm {\cos}\beta } )$. In our case $\alpha + \beta \ = \ \pi $, as *θ*_3_ = 0. Thus, ${R_1}\! ( {\rm {\cos}{\theta _1} - \rm {\cos}\alpha } )\! =\! {R_2}\! ( {\rm {\cos}{\theta _2}\, +} {\rm {\cos}\alpha } ) \Rightarrow {\gamma _1}\! ( {\rm {\cos}{\theta _1} {-} \rm {\cos}\alpha } )\, {=}\, {\gamma _2}\! ( {\rm {\cos}{\theta _2} + \rm {\cos}\alpha } ) \Rightarrow \rm {\cos}\alpha \ = \frac{{\gamma \rm {\cos}{\theta _1} - {\gamma _2}\rm {\cos}{\theta _2}}}{{{\gamma _1} + {\gamma _2}}}\ $. Finally we get

(3) \begin{equation*}{\theta _{app}} = \ \pi - \alpha \Rightarrow \rm {\cos}\ {\theta _{app}} = \frac{{{\gamma _2}\rm {\cos}{\theta _2} {-} {\gamma _1}{\rm cos}{\theta _1}}}{{{\gamma _1} + {\gamma _2}}}.\end{equation*}

Here *θ*_1_ is equal to the receding contact angle of a PDMS drop surrounded by water, and *θ*_2_ is the advancing contact angle of PDMS on the dry surface. For the three cases mentioned above we get the following values:

- On fluorinated glass, we measured *θ*_1_ ≈ 15° and *θ*_2_ = 50°. Then ${\theta _{app}}$= 120.6°.
- On stiff PDMS we get *θ*_1_ = 0 and *θ*_2_ = 0, so ${\theta _{app}}$ = 115.6°.
- At the boundary from slippery (i.e. PDMS) to hydrophobic surface, we have *θ*_1_ of the slippery side and θ_2_ of the hydrophobic (Fig. S8), thus *θ*_1_ = 0° and *θ*_2_ = 50°. Then ${\theta _{app}}$= 122.3°.

Based on the above analysis, the contact angle at the boundary of the heterogeneous surface is about 2° higher than the one on the homogeneous surface, which is in qualitative agreement with the experiments.

### Application of the SSS in drop impact

Maintaining a high energy recovery and a stable state without undergoing a wetting transition in the drop impact process is of importance in a variety of applications [31,32]. Here we verified the ability of the SSS for restitution and delaying wetting transition by drop impacting at a series of Weber numbers ($We\ = \ \rho {v^2}D{\gamma ^{ - 1}}$; *ρ*: liquid density; *v*: impact velocity; *D*: drop diameter; *γ*: water surface tension). The process of a drop impacting on SHPOS or SLIPS has been studied extensively recently [31,33–35]. It was generally found that the air layer entrapped between the impinging drop and underlying liquid or microstructure had a significant effect on the outcome of drop impact [33,35]. However, to the best of our knowledge, there was no report associated with the impact behavior of drops on the slippery SHPOS. To confer the role of the oil layer on the SSS in recovering the energy and stabilizing the microscopic water/air contact line during dynamic infiltration, we investigated the impact process on the control surface and the SSS.

**Figure 6.**
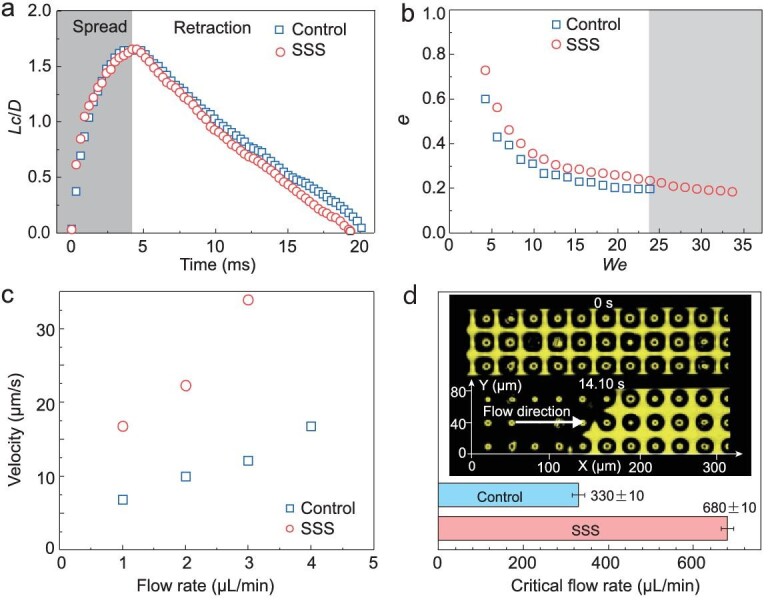
Application of SSS in drop impact and drag reduction. (a) Time-resolved variation of spread factor *Lc/D* (*Lc* is droplet contact length and *D* is the droplet diameter) on the control surface and SSS. (b) The restitution coefficient *e* as a function of Weber number *We*. (c) The velocity of tracers at the interface as a function of flow rate. The sizes of the structures used in the drop impact experiment are *d* = 15 μm, *D* = 10 μm, *b* = 30 μm, *h* = 22 μm, *H* = 26 μm. (d) Water/air interface impalement process at critical flow rate. The yellow color area in the images corresponds to the water/air interface on top or the reflection artifacts (yellow-black circle), and the black color indicates absence of reflection and thus corresponds to the water/solid interface. The sizes of the structures used in the drag reduction experiment are *d* = 15 μm, *D* = 10 μm, *b* = 30 μm, *h* = 16 μm, *H* = 20 μm.

Drop impacting both the SSS and the control surface showed complete rebound under certain conditions, similar to SHPOS [31]. Selected snapshots of a drop impact at *We* = 16.8 are shown in Fig. S11a and b. The corresponding time-resolved variation of spread factor *Lc/D* on the two surfaces is shown in Fig. 6a. Here, *Lc* is droplet contact length and *D* is the droplet diameter. Drops spread and exhibited a pancake shape at about 4.2 ${\rm{ms}}$ from contact with the surfaces. Then, they contracted laterally and finally bounced off the surface at about 19.4 ${\rm{ms}}$ for the SSS (Figs S11b and 6a, Video S6). The contact time (τ), measured as the time during which the drop was in apparent contact with the substrate, followed the scaling law of ${\rm{\tau }}\sim{({\rm{\rho }}{D^3}{\gamma^{ - 1}})^{\frac{1}{2}}}$ [34] with the fitting coefficient 1.0134 (Fig. S11c). This is consistent with theoretical analysis of SHPOS indicating that τ is not disturbed by the soft and slippery PDMS hemisphere. On the control structure, the drop required more time to detach from the surfaces, that is, 20.1 ${\rm{ms}}$ compared with 19.4 ${\rm{ms}}$ for SSS (Figs S11a and 6a). We attribute the difference to the viscous dissipation of energy on the surfaces [36], which means that the liquid film trapped in the inter-micropillar gaps is more easily detached and recovered under the action of lubricant on the top of the PDMS hemisphere. The little drop (Fig. S11a, red dashed frame) left on the control structure during the retraction also proved that water goes deeper, so the viscous force of the control structure was greater than that of the SSS.

By plotting the variation of the restitution coefficient (e) as a function of *We* (Fig. 6b), we found that the restitution coefficients of the two structures are slightly different under the whole range of given Weber numbers, namely ${{\rm{e}}_{SSS}}$ > ${e_{control}}$. The overall energy dissipation *E* of an impacting drop on the structures can be estimated by $E \sim We( {1 - {e^2}} )$ [35,36]. Therefore, the lubricating oil layer on the SSS made the water/air contact line more slippery. Besides, the Weber number ranges in which the drop can rebound on the two structures are significantly different as well, namely SSS > control, in accordance with the aforementioned higher stability of the SSS.

### Application of the SSS in drag reduction

The stable and mobile water/air contact line as exhibited by our SSS is necessary for drag reduction with stable Cassie-Baxter state such as in the inner wall of fluid pipelines. The drag reduction effect on SHPOS has been studied extensively recently [37–39]. We used microfluidic cells as a model of real-world applications of drag reduction and conducted flow measurements to quantitatively compare the velocity and the critical flow rate on the control surface and the SSS (Fig. S12a, Supplementary methods). Following the design of David Schäffel and co-workers, which obtained the flow velocity profiles on the topographical surfaces by fluorescent correlation spectroscopy (FCS) [37], we used total internal reflection fluorescent microscope (TIRFM) to observe directly the motion of the microspheres close to the interface and obtain the average velocity. We monitored the air cushion by confocal microscopy and determined the critical flow rate at which the microscopic water/air contact line depinned and moved to the Wenzel state (Fig. S12a). By analyzing the flow velocity and the critical flow rate, we compared the liquid mobility and the stability of the Cassie state on these two surfaces.

According to the interface slip theory, both the liquid/air interface and liquid/oil interfaces may show nonzero slip under certain flow rate (Fig. S12b) [37,40,41]. Compared to the control surface, which has slip on the liquid/air interface but zero slip at the liquid/solid interface, the SSS could have nonzero slip length at both liquid/air and liquid/oil interfaces and therefore larger drag reduction. To confirm this, we compared the average velocity of the microspheres flowing close to the protrusions of the SSS and the control surface at the same flow rate (Video S7). From Fig. 6c, it can be seen that the velocity on the SSS is 26% larger than that on the control surface at a flow rate of 1 μL/min (for calculation details see Figs S13–S16). As the flow rate increased, the amplitude of the difference became larger, reaching to 80% more than that on the control surface at 3 μL/min. This indicates that the drag reduction effect was improved on the SSS.

To compare the stability between the control surface and the SSS, we used confocal microscopy to measure the critical flow rate before impalement. At the critical velocity, the water/air interface disappeared gradually along the flow direction (Fig. 6d, Video S8). The critical flow rate was 680±10 μL/min for the SSS and 330±10 μL/min for the control surface. As the flow in the channel was driven by pressure, the higher flow rate indicated the higher pressed applied. Our results show that the SSS endured larger pressure than that of the control surface.

Consequently, the microfluidic experiment demonstrated that the molecular-smooth silicone oil layer on the SSS qualified the surface for slippery property and stability. The above two special features enable a longer lasting drag reduction effect in the pipeline with SSS structure. Additionally, the experiments with the microfluidic cell provided an important reference value for application of the structure to actual fluid pipelines.

## CONCLUSION

In conclusion, we have developed a facile method to fabricate a slippery SHPOS with enhanced stability. Using a modified dip-coating technique to create PDMS overhang micropillar arrays followed by the trap of silicone oil as a lubricant layer, we combined both superhydrophobic and slippery properties into one structure. The method is novel, simple and cost-effective, without the need for precise control, robotic devices or special training, as the final morphology is achieved by the effect of gravity and capillary forces. The lubricant creates an additional energy barrier, against quasi-static and dynamic impalement. Furthermore, the oil layer on the top of the structure also works as a lubricant which reduces the adhesion and improves the drop mobility significantly. Finally, we demonstrated stability against drop impact and enhanced drag reduction. We expect that the surface will be used in transport of viscous fluids, pipelines and microfluidic devices.

## METHODS

### Fabrication of the control surface and the SSS

The superhydrophobic micropillars were fabricated by the method as reported by Schellenberger *et al.* [24]. Polydimethylsiloxane (PDMS, mixture of Sylgard 184 elastomer kit and curing agent, ratio 10:1) was used to create the sphere head on the micropillars. A ${\rm{20 \pm 3\ \mu m\ }}$thick PDMS film was first coated on a cover glass by spin coating (12 000 rpm for 20 s), and transferred onto the top of the pillars by pressing them until full contact. After peeling off and turning over, the micropillars with PDMS heads were cured in a dryer at a temperature of 65°C for 4 hours. From the above process, the surface obtained is control surface. To make the PDMS hemisphere slippery, the surface was immersed in silicone oil (Sigma-Aldrich, viscosity 10 cSt) for 48 hours. Excess lubricant between the pillars was washed out with methylbenzene.

### Fabrication of model flat surfaces

The homogeneous hydrophobic surface was fabricated by fluorinating a bare cover glass using trichloro (1H, 1H, 2H, 2H-perfluorooctyl) silane by chemical vapor deposition (CVD). The heterogeneous surface consisted of a slippery circle of $3 \pm 0.5{\rm{\ mm\ }}$diameter in the center of a fluorinated hydrophobic surface. To obtain this surface, a bare cover glass was first activated by O_2_ plasma. Then, a circular PVC membrane with a diameter of $3 \pm 0.5{\rm{\ mm\ }}$was used as a mask to cover the center of the cover glass. After fluorination, the PVC membrane was removed and the hydrophilic circle was wetted with silicone oil. To measure advancing contact angles, a 13${\rm{\ \mu L}}$ water drop was deposited on the center of the slippery circle, forming a static apparent contact angle of $116 \pm 1^\circ $. Then, we increased the volume with a syringe pump at a rate of 2${\rm{\ \mu L}}/{\rm{min}}$. The process was recorded with use of a high-speed camera (Photron, Fastcam SA5) at a frame rate of 70 fps.

### Contact line receding process measurement by confocal microscopy

A laser scanning confocal microscope (NIKON A1, 20 ×/0.85 dry objective) with a resolution of about 0.62 ${\rm{\mu m}}$ and 1.0 μm in the lateral and axial direction of the image (Reflection mode) was used to record the receding side of water drops sliding on the control surface and the SSS (*d* = 15 μm, *D* = 10 μm, *b* = 60 μm, *h* = 16 μm, *H* = 20 μm, symbols explained in Fig. 2). A microneedle controlled by Micro Drive Stepper Motor Stage (ZOLIX, MAR100-90, China) was used to move the drop at a constant speed of 0.01 ${\rm{mm}}$/s. Images and videos were processed using the freely available ImageJ package [24,42].

### Drop evaporation experiment

Evaporation of 4 μL water drops on the control surface and SSS (structural size: *d* = 36 μm, *D* = 25 μm, *b* = 120 μm, *h* = 33 μm, *H* = 45 μm) was carried out at room temperature (24°C) at a relative humidity of 40%. The drop volume and the contact angle were recorded in real-time via the Data-Physics OCA 50 contact angle system. To measure the position of the interface between the drop and the air cushion during the evaporation-induced Cassie-to-Wenzel-state transition, we employed laser scanning confocal microscopy. Reflection mode was used for the measurement and the image was rebuilt as a 3D model using ‘Depth Code Alpha’.

## Supplementary Material

nwaa153_Supplemental_Files
