# Supplementary material for: Salvinia-like slippery surface with stable and mobile water/air contact line
Source: Natl Sci Rev. 2020 Jul 2;8(5):nwaa153. doi: 10.1093/nsr/nwaa153 (PMC8288347; doi:10.1093/nsr/nwaa153)
Supplement: nwaa153_Supplemental_Files [file nwaa153_supplemental_files.zip › NSR_MS-2020-442.R1-SI.docx]

Supplementary Information

***Salvinia*-like slippery surface with stable and mobile water/air contact line**

Xiaomei Li^1^, Jinlong Yang^1^, Kaixuan Lv^2^, Periklis Papadopoulos^3,6*^, Jing Sun^4^, Dehui Wang^1^, Yanhua Zhao^4^, Longquan Chen^5^, Dapeng Wang^2^, Zuankai Wang^4^ and Xu Deng^1*^

**Supplementary video** **captions**

**Video S1. Contact line receding process of water drop on the control surface and the SSS.** The structural dimensions are consistent on both surfaces: *d*=15 μm, *D*=10 μm, *b*=60 μm, *h*=16 μm, *H*=20 μm. The needle was kept at constant speed of 0.01 μm/s. The water/air and substrate/air interfaces (yellow) were imaged in reflection using laser scanning confocal microscopy (NIKON A1, 40 × dry objective). The micropillars of both SSS and the control surface were simulated. Real time: 7.096 min. Size: 318.198 × 58.5 μm².

**Video S2. Lateral adhesion force measurement of water drop on the control surface and the SSS.** A 4 μL water drop was deposited on the surface with the cantilever-based force sensor stuck in the drop. The surface underneath the drop was moved at constant velocity (*v*=0.43 mm/s), which was controlled by a Micro Drive Stepper Motor Stage (ZOLIX, MAR100-90, China). The whole process was recorded at 50 frames per second by a high-speed camera (Photron, Fastcam SA5).

**Video S3. Evaporation process of water drop on the control surface and the SSS recorded by a high-speed camera**. Water drop evaporated on the SSS (right) and control surface (left) recorded by a high-speed camera (Photron, Fastcam SA5, 2.5 x). The volume of drop is 4 μL and the size of the structure is: *d*=36 μm, *D*=25 μm, *b*=120 μm, *h*=33 μm, *H*=45 μm. The experiment condition is at temperature of 25°C and relative humidity of 40%. The video was recorded at 20 frames per second.

**Video S4: Evaporation process of water drop on the control surface and the SSS recorded by LSCM.** Water drop evaporated on the SSS (right) and the control surface (left) recorded by laser scanning confocal microscopy (NIKON A1, 20 × dry objective) in reflection mode. The volume of drop is 1 μL and the size of the structure is: *d*=36 μm, *D*=25 μm, *b*=120 μm, *h*=33 μm, *H*=45 μm. The experiment condition is at temperature of 25 °C and relative humidity of 40%. The real time is 9.34 min and the size is 633.77 μm x 633.77 μm x 87 μm.

**Video S5: Drop spreading on the chemically homogeneous and heterogeneous surfaces.** A water drop (13 μL) was deposited on the chemically homogeneous hydrophobic surface and heterogeneous slippery-hydrophobic composite surface. By injecting water (1 μL/min) into the drop, the contact line started to move after exceeding the critical apparent advancing angle. The video was recorded at 68 frames per second by a high-speed camera (Photron, Fastcam SA5, 2.5 x).

**Video S6. Impact process of water drop on the control surface and the SSS.** A Water drop impacted on the SSS (right) and the control surface (left). The sizes of the structures are: *d*=15 μm, *D*=10 μm, *b*=30μm, *h*=22 μm, *H*=26 μm. The Weber number is 16.8 and the drop diameter is 2.308 mm. The whole process was recorded at 5000 frames per second by a high-speed camera (Photron, Fastcam SA5).

**Video S7. Flowing process of microspheres close to the control surface and the SSS.** The process was recorded at by total internal reflection fluorescence microscopy (Nikon Ti-E, 100× oil immersion objective, 640 nm laser, 0.03 s acquisition time per image). The flow rate of liquid was 2 μL/min and the hydrodynamic radius of the microspheres is 195nm.

**Video S8. Impalement process at critical velocity in the micro-flow channel.** The process was recorded by laser scanning confocal microscopy (NIKON A1, 20 × dry objective) in reflection mode, the yellow color in the video is the water/air interface or reflection artifacts (small circle dots) and the array of dark circles shows the location of the pillars. Size: 318.198×80 μm². The sizes of the structures are: *d*=15 μm, *D*=10 μm, *b*=30μm, *h*=22 μm, *H*=26 μm.

**Supplementary methods**

**Lateral adhesion force measurement.** The lateral adhesion force of drops was measured by the deflection of a microneedle (diameter: 0.09mm, length: 75mm) that served as a cantilever-based force sensor. A 4 μL water drop was suspended at the lower tip of the microneedle. The drop was brought to contact with various surfaces that moved at a controlled speed. The process was record by a high-speed camera (Photron, Fastcam SA5) with a frame rate of 70 fps and analyzed by Tracker (A free video analysis and modeling tool built on the Open Source Physics (OSP) Java framework). The lateral adhesion force was linearly proportional to the deflection of the microneedle, and can be calculated by the elastic deflection according to the end-loaded cantilever beams deflection equation ($F=k\delta$, in which, *F* is lateral adhesion force, *k* is effective spring constant and $\delta$ is elastic deflection). Calibration was carried out with drops of known volume, giving *k* = 7.1 mN/m (Figure S4a). The dimension of the surface we used were: *d*=15 µm, *D*=10 µm, *b*=60 μm, *h*=16 μm, *H*=20 μm.

**Critical impalement pressure measurement.** Impalement pressure was measured using a high-sensitivity microelectromechanical balance system (Mettler Toledo, ML204). The surface was placed onto the balance and then a 4 µL water drop was placed on it. To increase the pressure applied by the drop to the surface, we contacted the top of the drop with a superamphiphobic plate and then moved down at the constant speed of 0.02 mm/s. Thus, the drop was squeezed between the test surface and the superamphiphobic surface. During this process, the force applied by the drop gradually increased until impalement occurred. The plate then continued to move down for 2 mm and then was withdrawn at the same speed. The dimension of the surface we used were: *d*=15 µm, *D*=10 µm, *b*=30 μm, *h*=16 μm, *H*=20 μm.

**Drop impact experiment.** Water drops (4 μL) impacted the surface (structural size: *d*=15 µm, *D*=10 µm, *b*=30 μm, *h*=16 μm, *H*=20 μm) from a height ranging from 0.5 mm to 50 mm. The drop impact dynamics was recorded by a high-speed camera (Photron, Fastcam SA5) with a frame rate of 10,000 fps and analyzed by ImageJ. All experiments were conducted in ambient environment at room temperature (24 ^o^C) and relative humidity of 50%.

**Micro-flow measurement.** The microchannel has a three-layer sandwich structure. The upper layer was either a control surface or SSS on a 1 mm thick microscope slide (25 mm x 60 mm) with two holes at both ends (diameter: 1 mm). The dimensions of the micropillar arrays were: *d* = 15 µm, *D* = 10 µm, *b* = 30 μm, *h* = 16 μm, *H* = 20 μm. The lowest layer of the construction was a microscope coverslip made of borosilicate glass (25 mm x 60 mm) with a thickness of 120 $\mu m$. The channel itself was created by cutting out a rectangular strip (2.5 mm x 50 mm) from an adhesive polymer film (3M, Scotch, double sided tape) with a thickness of 80 µm. The two holes on the upper slide were used to connect the channel with the inlet-outlet tubing. To make the structure water-proof, the three-layer construction as well as the inlet-/outlet- tubing was finally sealed by adhesive polymer (DEVCON, 5 minutes epoxy, 14265).

The critical flow rate was determined by confocal microscopy (Nikon A1). Reflection mode was used to scan the liquid/gas interface in the microfluidic channel. Under the continuous increase of the input flow rate, the wetting state is determined according to the existence or not of the liquid/gas interface.

To observe the local flow profile close to the pillars, we used tracer particles (Duke Scientific, hydrodynamic radius *R*_h_ = 195 nm) in deionized water. The microspheres were fluorescent and excited by a laser at 640 nm. The working fluid was pumped into the microfluidic channel from the inlet tube by a syringe pump (Harvard Apparatus, Pump 11 Pico Plus Syringe Pumps), at 5 different flow rates: 1 μL/min, 2 μL/min, 3 μL/min, 4 μL/min, and 5 μL/min. To improve the accuracy of the measurements and separate the contributions of convection and diffusion, the diffusive properties of the tracers were characterized and verified for each experiment. After the working liquid pumped into the microchannel, it was left to rest for 1h to stop convection. Then, we recorded the pure diffusion process to obtain the diffusion coefficient. After applying flow with the syringe pump, the presence of the strongly reflecting water/air interface, characteristic of the Cassie state, was checked by the self-focusing function of TIRFM, as well as the trajectories of single molecules. The single-molecule trajectories under flow at the surface of the microstructure were recorded using a Nikon Ti-E total internal reflection fluorescence microscope (TIRFM) with a 100× oil immersion objective. All trajectories were recorded at a horizontal plane 1 µm away from the micropillars.

**Supplementary figures**


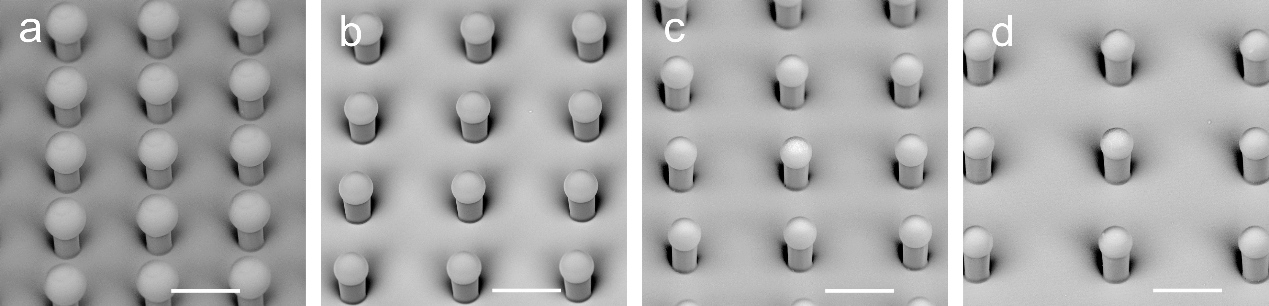


**Figure S1.** **SEM images of the control surfaces with different solid fraction.** (a) Structural size: *d*=15 μm, *D*=10 μm, *b*=30 μm, *h*=16 μm, *H*=20 μm, *f*=19.63%. (b) Structural size: *d*=15 μm, *D*=10 μm, *b*=40 μm, *h*=16 μm, *H*=20 μm, *f*=11.04%. (c) Structural size: *d*=15 μm, *D*=10 μm, *b*=50 μm, *h*=16 μm, *H*=20 μm, *f*=7.07%. (d) Structural size: *d*=15 μm, *D*=10 μm, *b*=60 μm, *h*=16 μm, *H*=20 μm, *f*=4.91%. The scale bar is 30 μm.


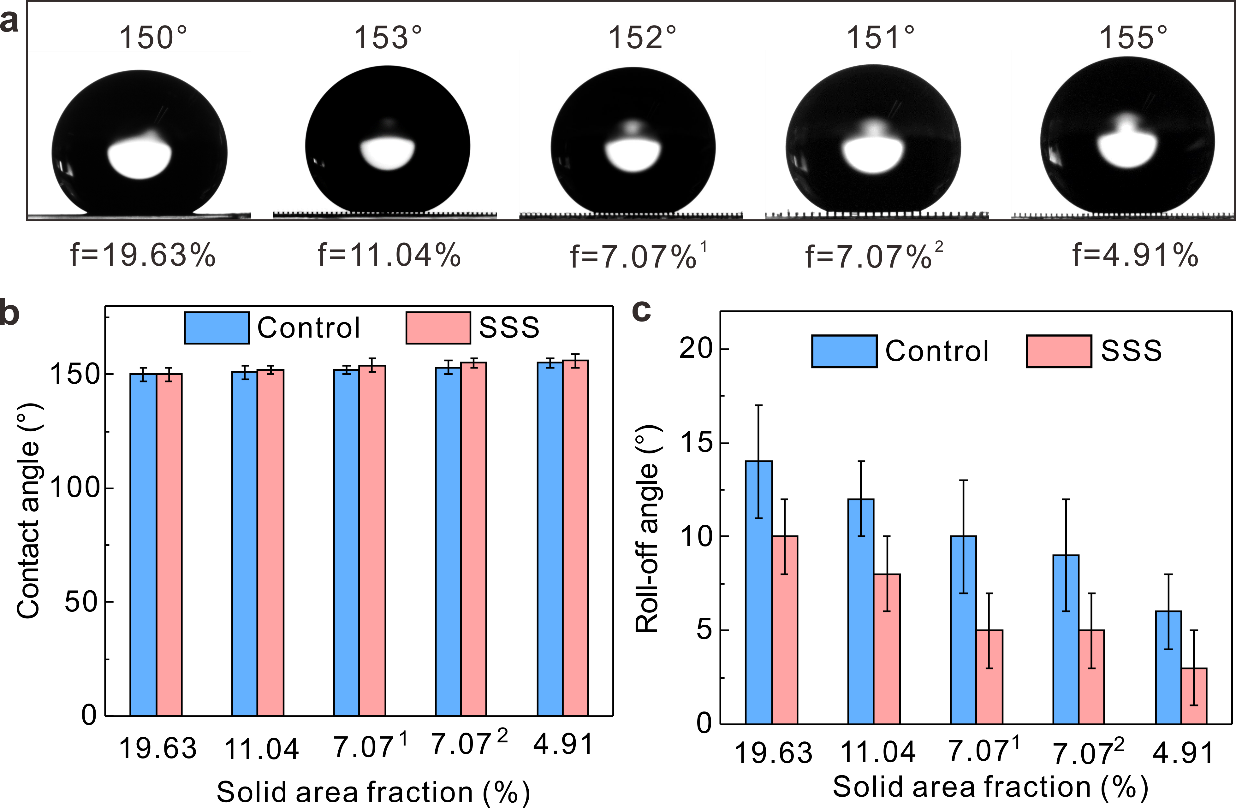


**Figure S2. Wetting of the control surface and the SSS.** (a) Apparent static contact angles of water drops on SSS with different solid fractions. (b) Apparent static contact angle of water drop (4 μL) on the SSS and the control surface with different solid fractions. (c) Roll-off angle of water drops (10 μL) on the SSS and the control surface with different solid fraction. Note: the structural size of *f*=7.07%^1^ is: *d*=15 μm, *D*=10 μm, *b*=60 μm, *h*=16 μm, *H*=20 μm, while the structural size of *f*=7.07%^2^ is: *d*=36 μm, *D*=25 μm, *b*=120 μm, *h*=33 μm, *H*=45 μm. Symbols as in Figure 2.


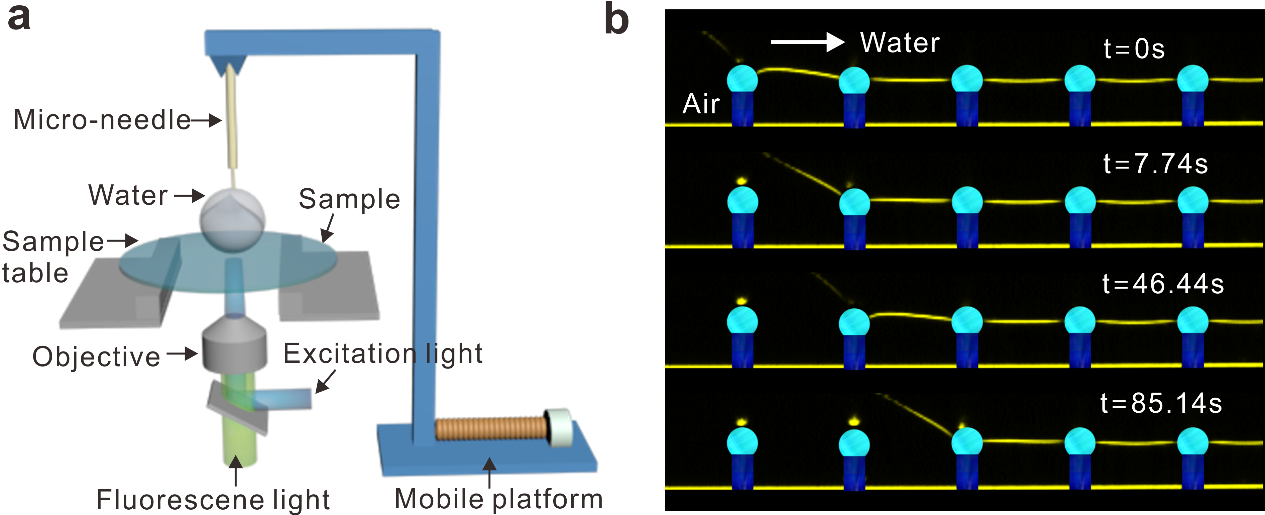


**Figure S3.** Confocal vertical cross section obtained from reflection signals (yellow) of the receding side of a water drop moving to the right on the control surface (*d*=15 μm, *D*=10 μm, *b*=60 μm, *h*= 16 μm, *H*=20 μm). Micropillars are simulated, with sizes measured by SEM.


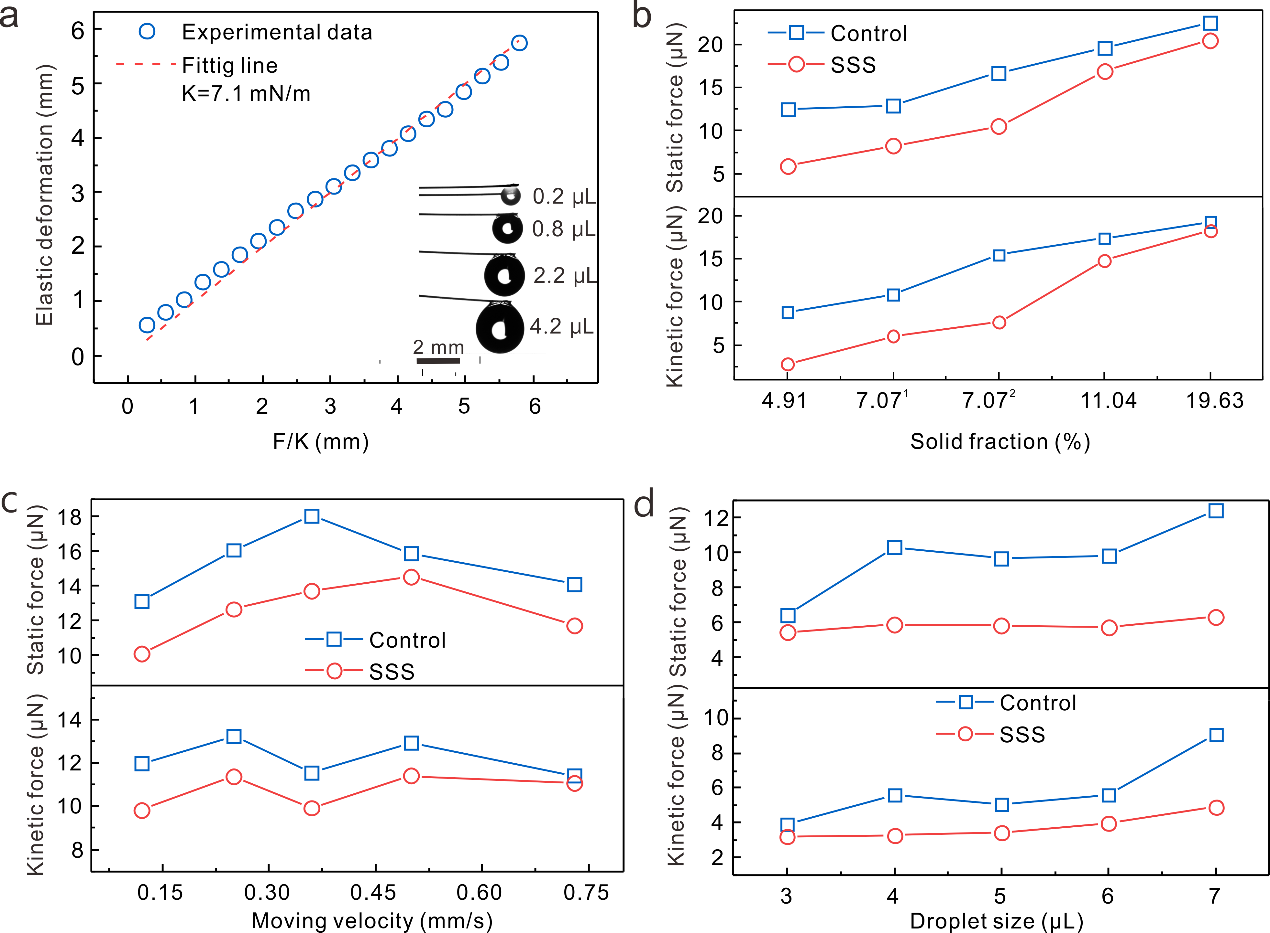


**Figure S4. Lateral adhesion force measurement.** (a) Standard curve of load force *vs* elastic deflection of the cantilever. The slope gives the constant *k* = 7.1 mN/m. The inset shows drops of known volume used for calibration. (b) Structural size effect on the lateral adhesion force for the control surface and the SSS. The structures are the same as in Fig. S2. (c) Speed effect on the lateral adhesion force for the control surface and the SSS. (d) Drop size effect on the lateral adhesion force for the control surface and the SSS.


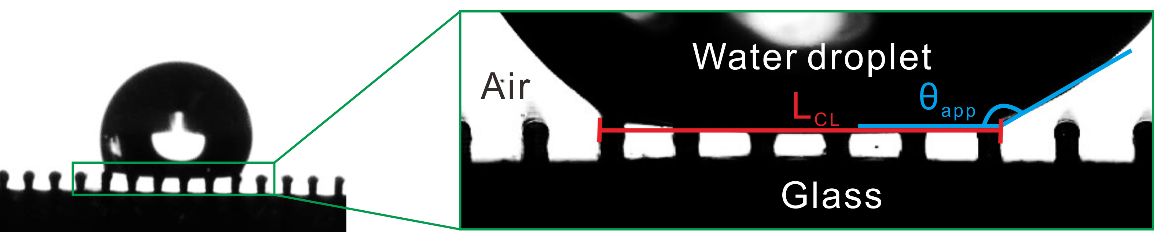


**Figure S5.** The definitions of the apparent contact angle $\theta_{app}$and the contact base diameter $L_{CL}$.


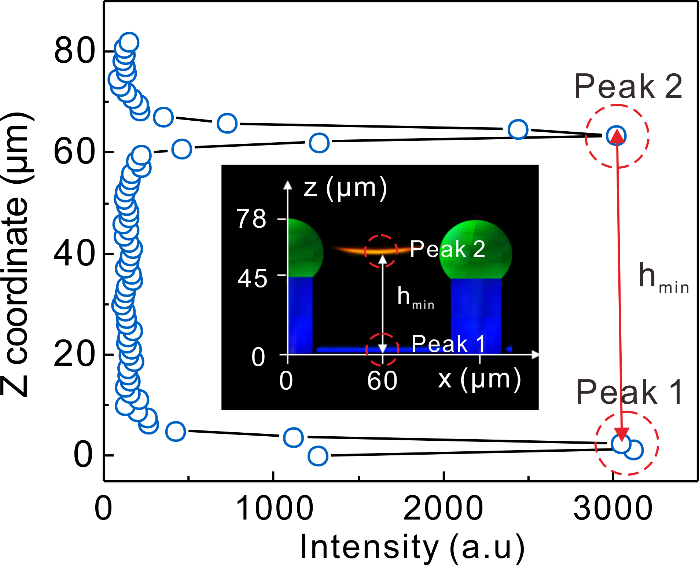


**Figure S6. Minimum thickness** $\boldsymbol{h}_{\boldsymbol{min}}$**of the air cushion.** The minimum thickness of the air cushion is in the middle between two pillars at the center of the drop, which is determined by the 3D contour confocal image. The value of the height is determined by the fluorescence intensity distribution curve in the Z direction (circles). The Z-direction spacing between the two intensity peaks (white arrow) is the thickness of air cushion.


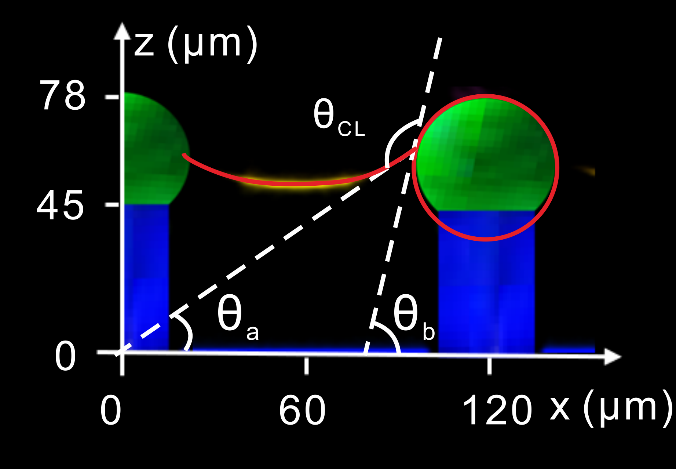


**Figure S7. Microscopic contact angle** $\boldsymbol{\theta}_{\boldsymbol{CL}}\boldsymbol{.}$ The microscopic contact angle is defined by the intersection of the water/air cushion (yellow) and PDMS hemisphere (green) interfaces. The water/air interface is curved, obeys the Young-Laplace equation and was imaged with the confocal microscope, up to a distance of 0.05d from the side wall of a pillar. The high refractive index of the pillars (*n* = 1.6) prevented measurement of the profile of the water/air interface closer to the walls. For the same reason PDMS and micropillars (blue) are simulated, with sizes known from SEM. The profile along the main axis of the microstructure arrays is fitted with the empirical equation *h* = *a*cosh(*x*/*b*) and is extrapolated to the edge of the pillars (red line). The hemisphere PDMS is fitting with the circle equation, $(x-60)^{2}+(z-60)^{2}={18}^{2}$, then the angle between the tangent lines of the two-equation curve at the intersection is the microscopic contact angle $\theta_{CL}$.


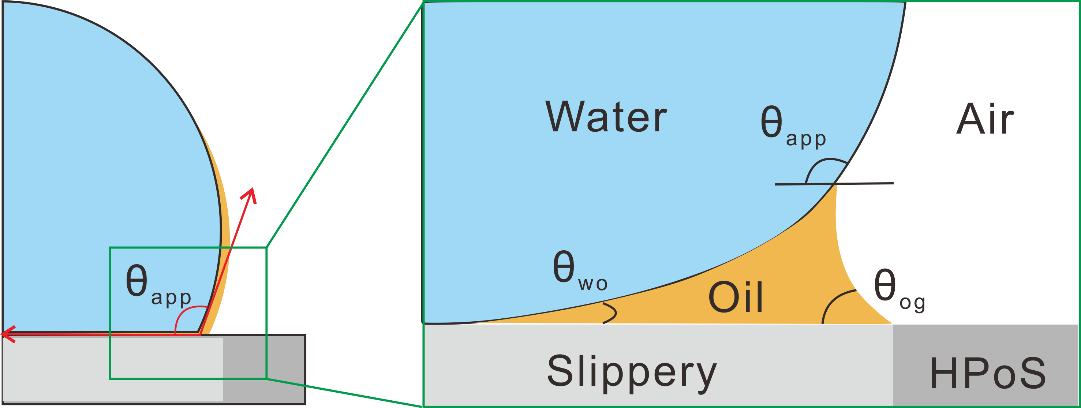


**Figure S8.** Sketch illustrating the geometry of a water drop (cyan) surrounded by a thin lubricant ridge (yellow) placed on a heterogeneous surface, composed of a hydrophilic slippery and a hydrophobic surface (HPOS). An energy, or alternatively contact angle, barrier was generated when the drop front advanced from the slippery to the hydrophobic region. *θ_wo_* is the material’s contact angles of oil surrounded by water, , and *θ_og_* is the contact angle of oil surrounded by air.


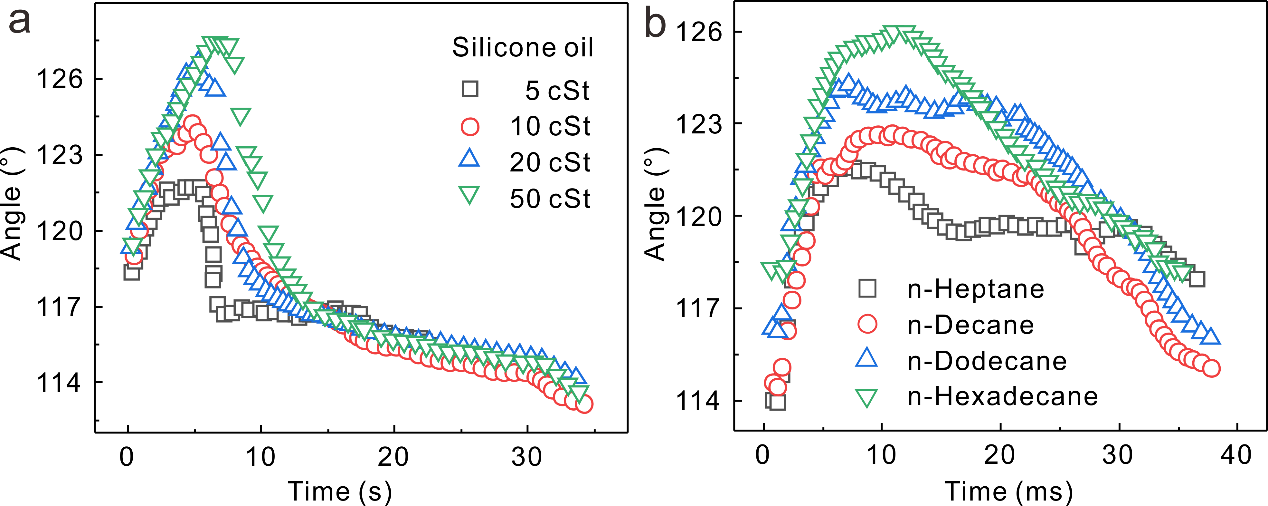


**Figure S9.** **Water drop spreading on chemically heterogeneous surface with different lubricants.** (a) Apparent contact angle of water as a function of time during spreading on the silicone oil/hydrophobic coating composite surface. (b) Apparent contact angle of water during spreading on the alkane/hydrophobic coating composite surface. Liquid properties are listed in Table S2.


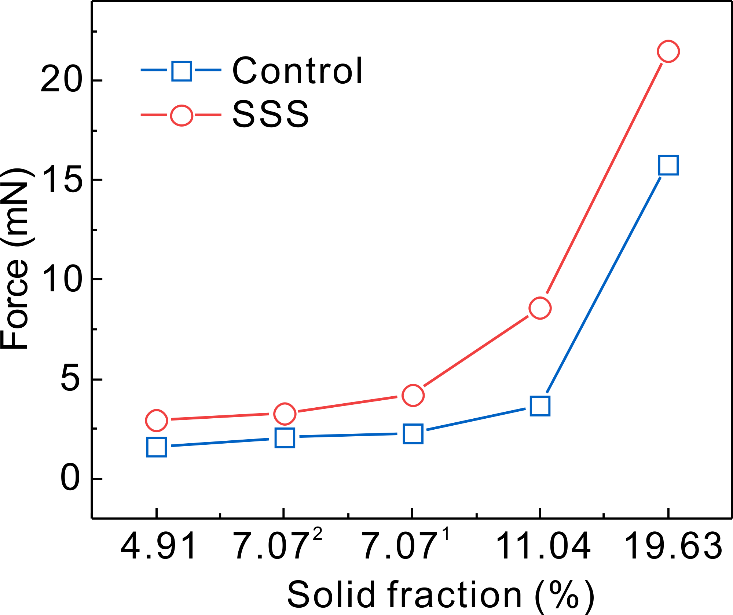


**Figure S10.** **Solid fraction effect on impalement force** **of the control surface and the SSS.** Note: the structural size of *f*=7.07%^1^ is: *d*=15 μm, *D*=10 μm, *b*=60 μm, *h*=16 μm, *H*=20 μm, while the structural size of *f*=7.07%^2^ is *d*=36 μm, *D*=25 μm, *b*=120 μm, *h*=33 μm, *H*=45 μm.


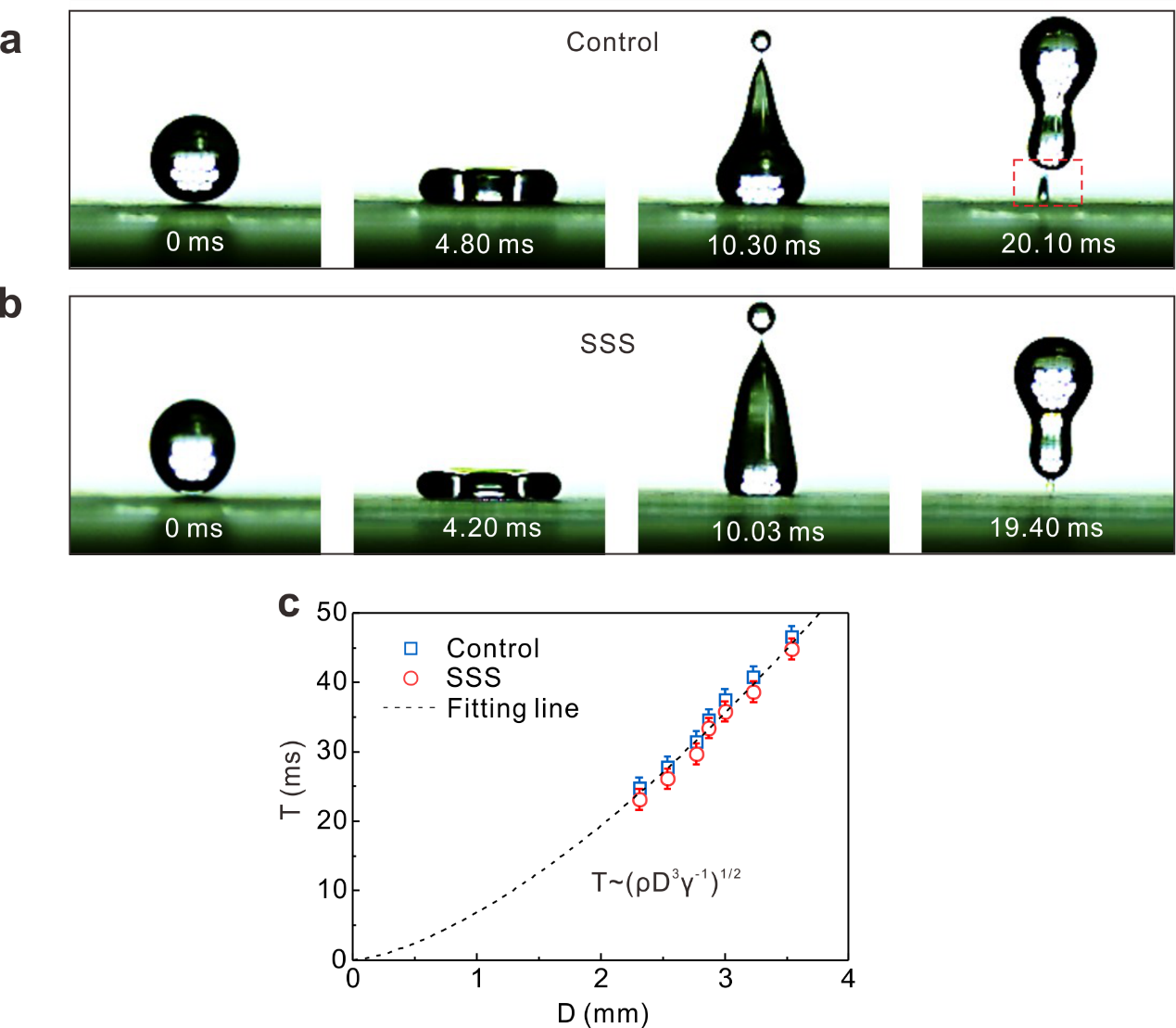


**Figure S11. Impact process of water drop on the control surface and the SSS.** (a) Selected snapshots show the complete rebound of drops impacting on the control surfaces and (b) the SSS (*We*=16.8, drop diameter: 2.308 mm). Red dotted square indicates the micro-drop which was pulled down by the control surfaces with higher viscous force during the rebound process. (c) Contact time of a bouncing drop follows the scaling law:$\tau\sim(\rho D^{3}\gamma^{-1})^{1/2}$, ^1^which is consistent with theoretical analysis on superhydrophobic surfaces, so the impact on the SSS also behaved superhydrophobic-like bouncing.


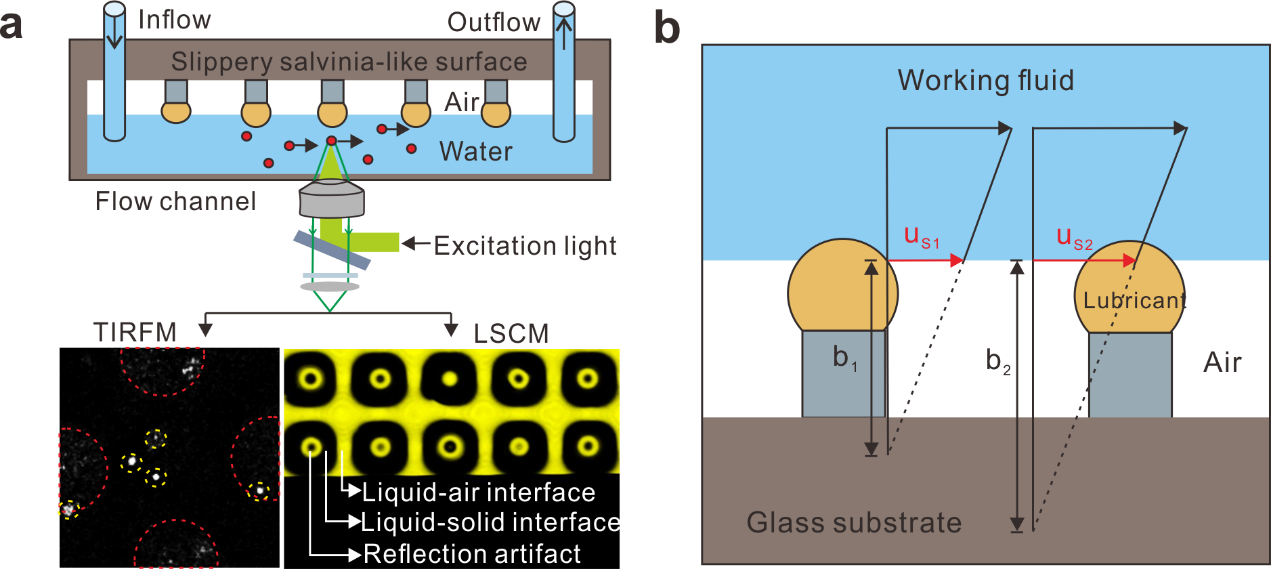


**Figure S12.** (a) Schematic of the experimental setup. TIRFM was used to record the motion of tracers (down left: Inside the yellow dotted circles are the tracers, and the red dotted circles are the microstructures), LSCM was used to confirm the impalement of water/air contact line (down right). (b) Schematic diagram of the drag-reduction effect on the SSS.


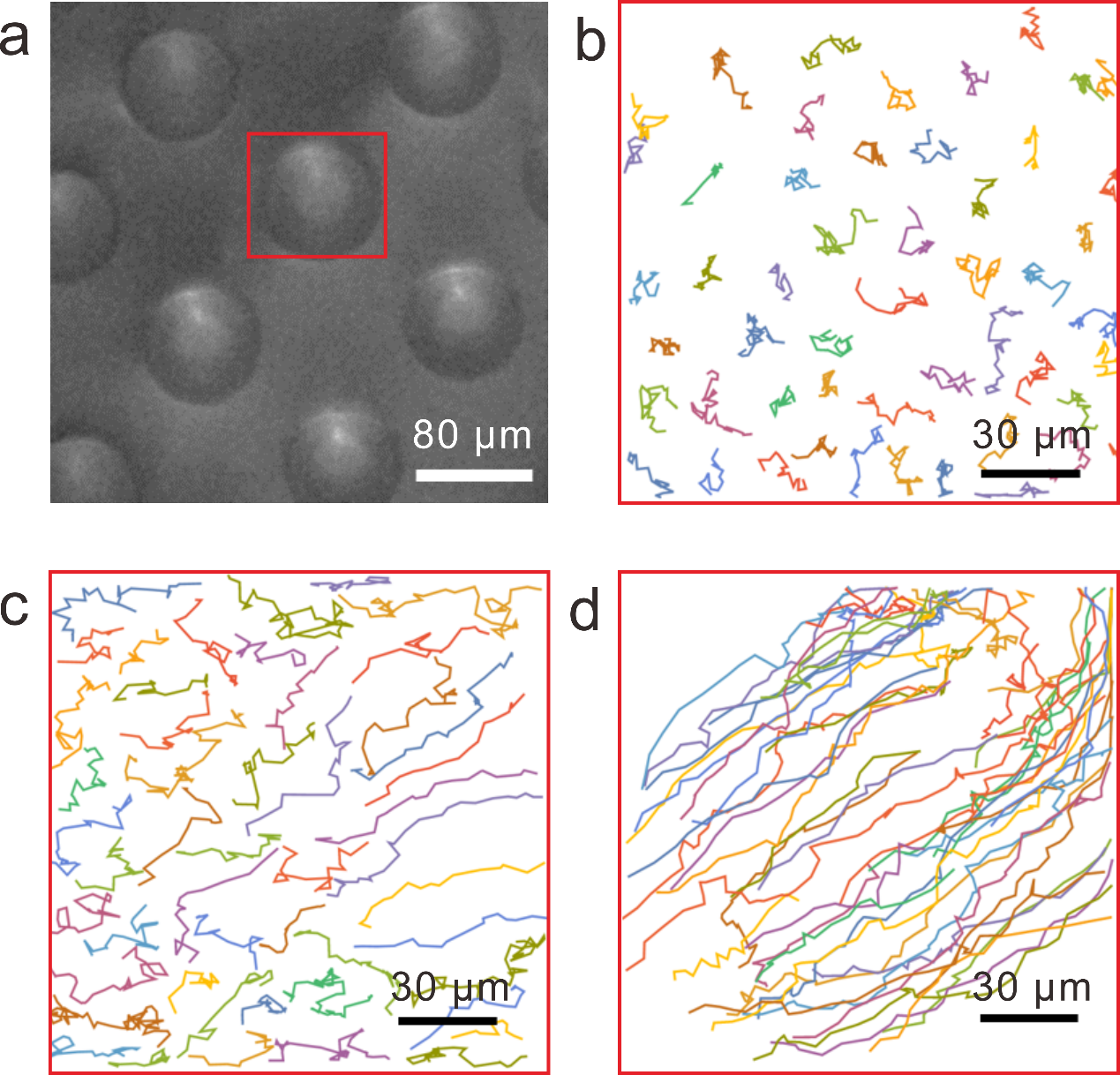


**Figure S13.** (a) Pillar arrays on the SSS imaged by TIRFM. The red frame is the observation region. (b) Representative trajectories of particles at the interface without flow rate on the control surface, (c) with flow rate of 1$\mu L/min$ on the control surface, and (d) with flow rate of 1$\mu L/min$ on the SSS, respectively.

Instead of using the micro-PIV directly, we used single-molecule TIRFM to track the motion of single tracer particles. The direct illumination (no TIRFM) was used to characterize the flow in our microchannel. As tracers, we used microspheres (Duke Scientific) with a hydrodynamic radius of *Rh* = 195 nm. The tracers were illuminated with a laser (640 nm) through a Nikon PL Apo NA 1.45 100x TIRF oil immersion objective at zero angle. The near-surface location was found by the automatic microscope focusing system. We finally focused at 1μm from the water/solid interface. Nikon Elements software was then used to capture the time series of images from the cooled CCD camera (Photometrics Cascade 512B). Continuous 1.7-minute image sequences were recorded with an exposure time of 0.03s. The number of tracers tracked during the experiment ranged from 600 to 1000 objects, depending on the illumination conditions and flow rate. A custom Mathematica program identified and tracked the tracers from the recorded series of images as previously described^2^.


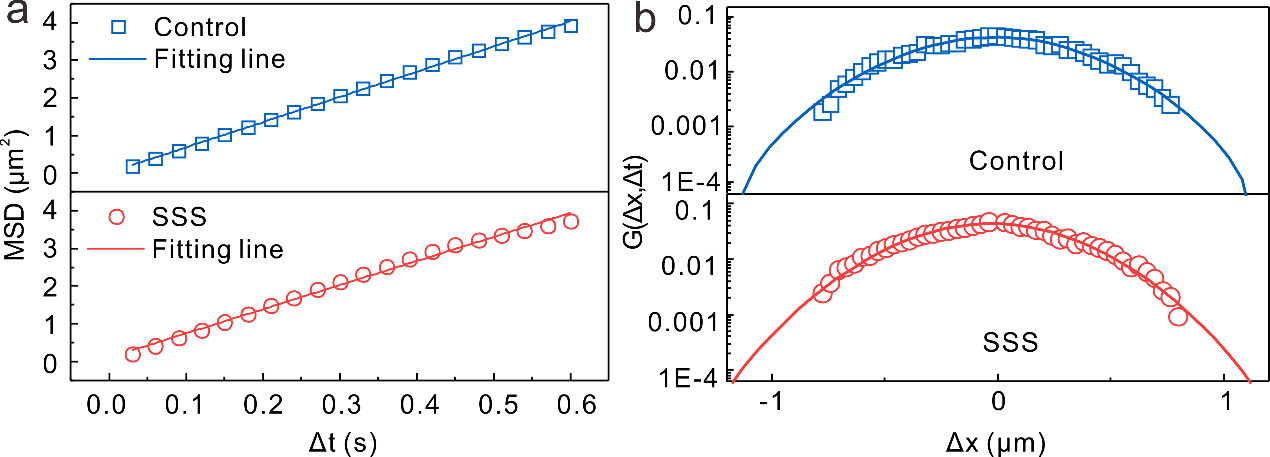


**Figure S14. Diffusion at the interface without flow.** (a) Mean-square displacement (MSD) *vs* lag time *Δt* for particles without flow on the SSS and the control surface. Symbols indicate experimental data, the solid lines represent linear fits by *MSD = 4DΔt*, where *D* is the diffusion coefficient. (b) Displacement distributions for particles without flow. The symbols denote experimental data. The solid lines represent Gaussian fits *G(Δx) = Aexp(−Δx^2^/2σ^2^)*, where A is the amplitude and *σ^2^* is the variance^3^.Without flow, the mean square displacements of particles are proportional to lag time for both two surfaces, and the displacement distributions are Gaussian distribution, indicating that the nanoparticles undergo Brownian motion on both surfaces. The diffusion coefficient of particles on the control surface is 1.674 μm^2^∙s^-1^, and on the SSS is 1.598 μm^2^∙s^-1^


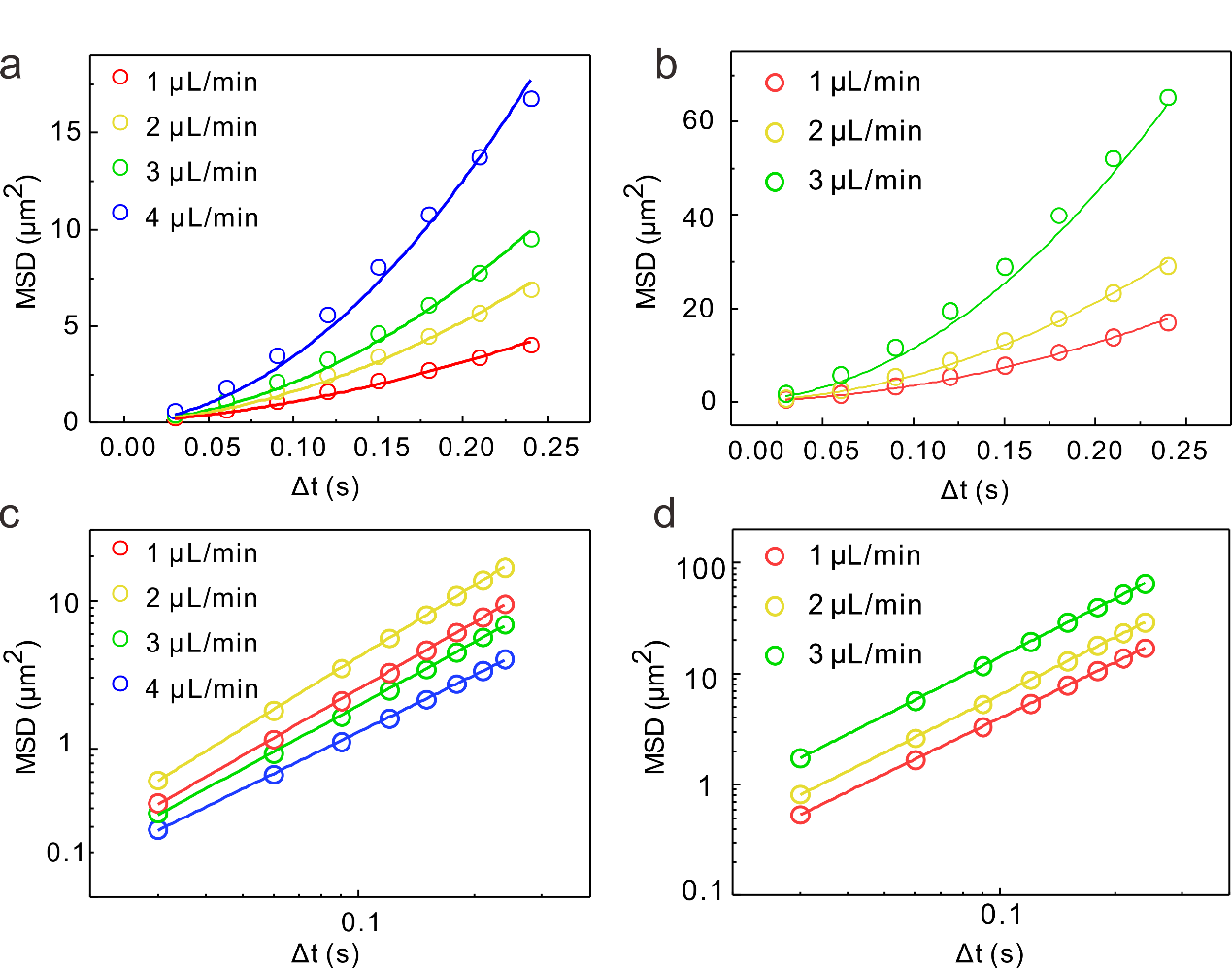


**Figure S15. MSD *vs* lag time for microspheres in various flow rates at pillar/water interfaces of the two surfaces**. (a) MSD on the control surface. (b) MSD on the SSS. Symbols indicate experimental data, the solid lines represent linear fits by *MSD = v^2^∆t^2^+4DΔt*. (c) Double logarithmic curve of MSD on the control surface, and (d) the SSS.


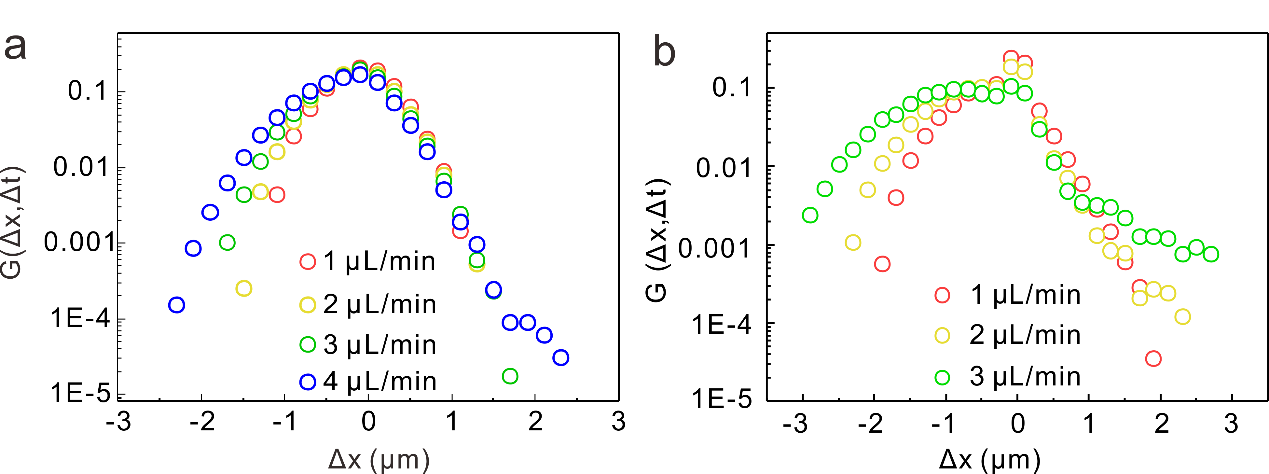


**Figure S16. Displacement distributions for particles in various flow rates at pillar-water interfaces of the two surfaces.** (a) The control surfaces. (b) The SSS. The symbols denote experimental data.

**Supplemental table**

Table S1. Advancing and receding angles of probe liquids on the fluorinated glass.

| Liquid | $\gamma$ (mN/m) | $\rho(\mathrm{cSt})$ | Adv ($^{\circ})$ | Rec ($^{\circ})$ |
| --- | --- | --- | --- | --- |
| n-Hexadecane | 27^4^ | 4^5^ | 85±5 | 49±2 |
| n-Dodecane | 25^4^ | 2^5^ | 76±3 | 47±3 |
| n-Decane | 23^4^ | 1.3^5^ | 58±3 | 43±5 |
| n-Heptane | 20^4^ | 0.6^5^ | 55±5 | <10 |
| Silicone oil 1 | 20^6^ | 5 | 54±5 | 33±5 |
| Silicone oil 2 | 20^6^ | 10 | 59±5 | 36±5 |
| Silicone oil 3 | 20^6^ | 20 | 55±5 | 35±5 |
| Silicone oil 4 | 20^6^ | 50 | 54±5 | 36±5 |

Note: advancing angles (Adv) and receding angles (Rec) were measured using a 10 μL water drop. Errors are standard deviations obtained from at least 5 independent measurements.

Table S2. Wetting of the flat films

| Sample | $\theta(^{\circ})$ | $\theta_{adv} (^{\circ})$ | $\theta_{rec} (^{\circ})$ | $\theta_{roll-off} (^{\circ})$ |
| --- | --- | --- | --- | --- |
| Flat fluorinated SU8 film | 118±1 | 120±3 | 84±1 | NR |
| Flat PDMS film | 104±1 | 115±2 | 88±1 | NR |
| Flat oil infused PDMS film | 100±2 | 110±2 | 100±2 | 10±1 |

Note: apparent contact angle was measured using a 4 $\mu L$ water drop. Advancing angle, receding angle and roll-off angle were measured using a 10 $\mu L$ water drop. “NR” means the water drop did not roll off the surface even when the surface was tilted exceed 90°. Errors are standard deviations obtained from at least 5 independent measurements.

**Reference**

1. Hao CL, Li J and Liu Y *et al.* Superhydrophobic-like tunable droplet bouncing on slippery liquid interfaces. *Nat Commun* 2015; **6**: 7986.

2. Walder R and Schwartz DK. Dynamics of protein aggregation at the oil–water interface characterized by single molecule TIRF microscopy. *Soft Matter* 2011; **7**: 7616-7622.

3. Wang DP, Hu RF and Mabry JN *et al.* Scaling of polymer dynamics at an oil-water interface in regimes dominated by viscous drag and desorption-mediated flights. *J Am Chem Soc* 2015; **137**: 12312-12320.

4. Pan SJ, Guo R and Björnmalm M *et al.* Coatings super-repellent to ultralow surface tension liquids. *Nat Mater* 2018; **17**: 1040-1047.

5. Aucejo A, Burguet MC and Munoz R *et al.* Viscosities, and refractive indices of some n-alkane binary liquid systems at 298.15 K. *J Chem Eng Data* 1995; **40**: 141-147.

6. Kreder MJ, Daniel D and Tetreault A *et al.* Film dynamics and lubricant depletion by droplets moving on lubricated surfaces. *Phys Rev X* 2018; **8**: 031053.
